# Temporal Variation of Mycotoxin Producing Fungi in Norwegian Cereals

**DOI:** 10.3390/microorganisms1010188

**Published:** 2013-12-17

**Authors:** Leif Sundheim, Guro Brodal, Inger S. Hofgaard, Trond Rafoss

**Affiliations:** 1 Norwegian Institute for Agricultural and Environmental Research, Fr. A. Dahlsvei 20, Ås 1430, Norway; E-Mails: guro.brodal@bioforsk.no (G.B.); ingerd.hofgaard@bioforsk.no (I.S.H.); trond.rafoss@bioforsk.no (T.R.); 2 Norwegian Scientific Committee for Food Safety, P.O. Box 4404, Nydalen, Oslo 0403, Norway

**Keywords:** *Fusarium*, deoxynivalenol, nivalenol, precipitation

## Abstract

Spring barley is grown on about half of the Norwegian cereal area. The rest of the area is equally divided between wheat and oats. Most years the domestic production provides 70%–80% of the domestic market for bread wheat. Barley and oats are mainly grown for animal feed. During the years 2008–2012, severe epidemics of *Fusarium* head blight have led to increased mycotoxin contamination of cereals. During that period, precipitation was above normal during anthesis and grain maturation. The most important mycotoxin producers have been *F. avenaceum*, *F. culmorum*, *F. graminearum* and *F. langsethiae*. Increased deoxynivalenol contamination of Norwegian cereals during recent years is due to severe *F. graminearum* epidemics.

## 1. Introduction

In Norway and Finland, cereals are grown further north than anywhere else. About 85% of the cereal production is concentrated in the south-eastern counties, while the majority of the remainder is located in the Trøndelag counties in central Norway [1]. Most cereal growers practice monoculture on farms without domestic animals. Spring barley is grown on about half of the 300,000 ha of cereals, and the rest of the area is equally divided between growth of wheat and oats. During most of the last ten years, domestic production has provided 70%–80% of the domestic bread wheat consumption. Barley and oats are mainly used for animal feed with less than 5% of the production for human consumption. A risk assessment of mycotoxins in Norwegian cereals was published in 2013 [2]. We summarize data on mycotoxin producing fungi in Norway during the last century.

## 2. The Most Prevalent *Fusarium* spp. in Norwegian Cereals

An increased occurrence of mycotoxin-producing *Fusarium* spp. has been reported in Norway over the last decade. The cereal harvests during the years 2008–2012 gave particular reason for concern, as precipitation was higher than normal during the period from anthesis to harvest, which is favorable for epidemic development of *Fusarium* spp. [2].

Most reports list *Fusarium avenaceum* (Fr.) Sacc. as the most common *Fusarium* species in Norwegian cereals [3,4,5] (Table 1, Table 2, Table 3, Table 4 and Table 5). It is widely distributed in temperate regions and is most commonly soil-borne [6]. *Fusarium avenaceum* can be isolated from cereal seeds, cereal foot rot, and causes head blight. The teleomorph stage, *Gibberella avenacea* Cooke, has not been found in Norway. Langseth *et al.* [7] reported that Norwegian strains of *F. avenaceum* produce moniliformin and enniatin.

**Table 1:** Occurrence (%) of *Fusarium* spp. isolated from symptomatic cereal plants in Norway from 1980 to 1983, as determined by Haave [3].

Isolated Species	Wheat	Barley	Oats	Mean
*F. avenaceum*	21.6	20.5	25.2	25.0
*F. culmorum*	25.3	25.6	38.2	29.6
*F. graminearum*	7.4	4.3	11.5	7.8
*F. poae*	2.5	0.9	-	1.2
*F. equiseti*	1.9	0.9	-	1.0
*F. tricinctum*	0.6	0.8	-	0.5

**Table 2:** Occurrence (%) of *Fusarium* spp. isolated from seed samples at five experimental fields in Norway from 1994 to 1997, as determined by Henriksen [4].

Field	Kvithamar	Apelsvoll	Brandval	Norderås	Hauer	Mean
County	N-Trøndelag	Oppland	Hedmark	Akershus	Akershus	
Isolated Species						
*F. avenaceum*	32.3	4.0	45.5	5.9	12.4	20.0
*F. culmorum*	1.4	0.2	7.5	1.5	2.2	2.5
*F. graminearum*	2.1	0.5	2.4	0	0	1.0
*F. poae*	5.4	6.3	4.0	13.0	10.7	7.9
*F. tricinctum*	3.3	10.2	1.2	2.5	32.3	9.9
*F. crookwellense*	3.0	0.05	5.5	0.6	0.2	1.9

**Table 3:** Occurrence (%) of *Fusarium* spp. isolated from wheat samples from four regions in Norway from 1994 to 1996, as determined by Kosiak *et al.* [5].

Isolated species	South-East	Upper-East	Mid-Norway	South-West
*F. avenaceum*	97.6	100.0	100.0	100.0
*F. culmorum*	65.9	78.0	28.6	68.8
*F. graminearum*	9.8	36.6	42.9	12.5
*F. poae*	87.8	95.1	42.9	85.4
*F. equiseti*	4.9	29.3	7.1	31.3
*F. tricinctum*	70.7	65.9	50.0	77.1
*F. sporotrichoides*	4.9	4.9	0.0	8.3
*F. langsethiae*	76.5	70.6	56.6	70.0

**Table 4:** Occurrence (%) of *Fusarium* spp. isolated from barley samples from four regions in Norway from 1994 to 1996, as determined by Kosiak *et al.* [5].

Isolated species	South-East	Upper-East	Mid-Norway	South-West
*F. avenaceum*	100.0	100.0	100.0	97.4
*F. culmorum*	77.8	69.2	54.9	82.1
*F. graminearum*	20.4	26.9	31.0	14.1
*F. poae*	94.4	83.3	43.7	91.0
*F. equiseti*	14.9	34.6	4.2	34.6
*F. tricinctum*	90.7	93.6	62.0	100.0
*F. sporotrichoides*	3.7	7.7	1.4	9.0
*F. langsethiae*	71.4	84.0	62.5	73.3

**Table 5:** Occurrence (%) of *Fusarium* spp. isolated from oat samples from four regions in Norway from 1994 to 1996, as determined by Kosiak *et al.* [5].

Isolated species	South-East	Upper-East	Mid-Norway	South-West
*F. avenaceum*	98.1	98.8	100.0	98.8
*F. culmorum*	72.2	77.5	45.5	81.5
*F. graminearum*	13.0	31.3	72.7	28.4
*F. poae*	96.3	90.0	49.1	96.3
*F. equiseti*	20.4	30.0	1.8	24.7
*F. tricinctum*	83.3	90.0	65.5	88.9
*F. sporotrichoides*	7.4	1.3	0.0	4.9
*F. langsethiae*	95.5	96.3	30.0	88.5

The frequency of *Fusarium crookwellense* L.W. Burgess, P.E. Nelson and Toussoun is low in Norway [4] (Table 2). *Fusarium crookwellense* strains produce fusarin C [8], zearalenone (ZON) and nivalenol (NIV). In a review, Schollenberger *et al.* [9] list *Fusarium crookwellense* among the fungi producing type A trichothecenes in the diacetoxyscirpenol (DAS) group.

One of the most commonly isolated fungi from Norwegian cereals has been *Fusarium culmorum* (W.G. Sm.) Sacc. [3,4,5] (Table 1, Table 2, Table 3, Table 4 and Table 5). However, during the last ten years the frequency of this species has decreased [5]. In temperate regions, *F. culmorum* causes seedling blight, foot rot and head blight of cereals, and the fungus is common on cereal plant debris. *Fusarium culmorum* contains the *tri5* and *tri6* genes for trichothecene biosynthesis [10]. Schollenberger *et al.* [9] include *F. culmorum* among those fungi producing DAS. Langseth *et al.* [7] reported that Norwegian strains of *F. culmorum* produced nivalenol (NIV), deoxynivalenol (DON), the acetylated DON-derivative 3-acetyldeoxynivalenol (3-ADON) and ZON. Bakan *et al.* [11] divided *F. culmorum* into two chemotypes, the NIV chemotype and the DON chemotype; and the authors reported that these chemotypes are distributed independently of wheat variety and geographical origin. ZON production was significantly higher in the DON-producing strains of *F. culmorum* than in the NIV-producing strains. DON appears to play a role in the pathogenicity of *F. culmorum* [12]. In Denmark, Hestbjerg *et al.* [13] found correlation between DON content and aggressiveness. The frequency of *Fusarium equiseti* (Corda) Sacc. with the teleomorph stage *Gibberella intricans* Wollenw. is low to medium in Norwegian cereals [3,5] (Table 1, Table 2, Table 3, Table 4 and Table 5). Schollenberger *et al.* [9] included *F. equiseti* among fungi that produce DAS. Langseth *et al.* [7] reported that Norwegian strains of *F. equiseti* produced DAS, NIV, and ZON.

*Fusarium graminearum* Schwabe has a long history as a pathogen of cereals in Norway and it is common in barley, oats and wheat [3,4,5] (Table 1, Table 2, Table 3, Table 4 and Table 5). During the last ten years the prevalence of *F. graminearum* has increased in Norway [5]. However, Roll-Hansen [14] reported that the fungus has been a cereal pathogen in Norway for more than 70 years. In Germany, Oerke *et al.* [15] concluded that *F. graminearum* is the only *Fusarium* species in which airborne sexual spores contribute significantly to dissemination and development of epidemics in cereals. Schollenberger *et al.* [9] reported that *F. graminearum* produces type A trichothecenes in the DAS group. Norwegian strains of *F. graminearum* produce 3-ADON and ZON [7,16]. Fusarin C is also produced by *F. graminearum* isolates [8]. Ward *et al.* [17] described three chemotypes of *F. graminearum*: (i) NIV chemotype that produces NIV and its acetylated derivatives; (ii) 3-ADON chemotype that produces 3-ADON; and (iii) 15-ADON chemotype that produces 15-ADON. In Northern Europe, the 3-ADON genotype has been dominant, while in Southern and Central Europe the 15-ADON genotype is the most prevalent [18]. DON is a pathogenicity factor for *F. graminearum* causing necrosis in wheat leaves, which allows the fungus to spread into the rachis from florets [19]. Waalwijk *et al.* [20] employed multiplex PCR and found *F. graminearum* to be the most abundant species in Dutch wheat fields during the years 2000–2001. This contrasts with results from earlier studies in 1980s and 1990s, when *F. culmorum* was more common than *F. graminearum* in the Netherlands. The authors suggested that increased cultivation of maize in the Netherlands and warmer summers were the factors behind the change. Increases in the prevalence of *F. graminearum* have been reported from several European countries [21].

Torp and Nirenberg in 2004 described *Fusarium* strains, previously known as “powdery” *F. poae*, as *F. langsethiae* [22]. In recent studies the frequency of *F. langsethiae* in Norway has been found to be medium to high [5] (Table 3, Table 4 and Table 5). This species is a common *Fusarium* sp. in oats in Norway [5]. Phylogenetic analyses have confirmed that *F. langsethiae* is a distinct, new species, and that *F. langsethiae* is more closely related to *F. sporotrichioides* than to *F. poae* [23]. Imathiu *et al.* [24] reported that *F. langsethiae* could be isolated from symptomless oat and wheat grain, but the authors were unable to demonstrate any evidence of pathogenicity in inoculation experiments on wheat leaves. When different inoculation methods for *F. langsethiae* in oats were compared, Divon *et al.* [25] found that the fungus has a strong preference for panicle infection. Thrane *et al.* [26] concluded that *F. langsethiae* produced enniatin and the type A trichothecenes T-2, HT-2 and DAS. Aamot *et al.* [27] confirmed the production of T-2 and HT-2 in oats and detected up to 2040 μg/kg in Norwegian oats sampled during the years 2004-2009.

The frequency of *Fusarium poae* (Peck) Wollenw. is low to medium in cereal grains in Norway [3,4,5] (Table 1, Table 2, Table 3, Table 4 and Table 5). However, isolates of the morphologically similar species *F. langsethiae* were probably included in *F. poae* until *F. langsethiae* was described as a separate species [22]. *Fusarium poae* is most common in temperate regions, and cereal seeds and heads are commonly contaminated by this fungus. Liu and Sundheim [28] identified 13 vegetative compatibility groups (VCG) in 22 Norwegian and two Polish isolates of *F. poae*. The relatively large number of VCG groups indicates that sexual recombination occurs infrequently in this species. Liu *et al.* [29] found that 20 of the 24 *F. poae* isolates from Norway and Poland produced DAS, and half of the isolates produced NIV. None of the isolates produced T-2 or HT-2 toxins. Langseth *et al.* [7] reported that of five Norwegian *F. poae* strains, one produced NIV and DAS and four strains produced ZON.

In Norway the frequency of *Fusarium sporotrichioides* Sherb. is low [3,4,5] (Table 3, Table 4 and Table 5). *Fusarium sporotrichioides* is widely distributed on cereals, grasses and alfalfa in temperate regions [30]. Phylogenetic analyses have confirmed that *F. sporotrichioides* and *F. langsethiae* are sister taxa [23]. Strains of *F. sporotrichioides* produce HT-2, T-2, DAS, fusarin C and enniatin [8,26]. Three Norwegian strains of *F. sporotrichioides* all produced T2, HT-2, DAS and ZON [7].

The frequency of *Fusarium tricinctum* (Corda) Sacc. is low to medium in Norway [3,4,5] (Table 1, Table 2, Table 3, Table 4 and Table 5) . Golinski *et al.* [31] concluded that the fungus is most common in temperate regions, and that *F. tricinctum* usually occurs as a saprophyte or a weak parasite. Langseth *et al.* [7] found that two of three Norwegian strains of *F. tricinctum* produced moniliformin, and all three produced traces of enniatin. *Fusarium tricinctum* is also known to produce fusarin C [6].

## 3. Temporal Variation of *Fusarium* Species

Over the years the importance of *Fusarium* species in Norwegian cereal crops has increased [2]. Drivers for these changes may have been the development of specialized cereal production regions and changes in weather conditions. During the last fifty years there have been dramatic changes in the cropping practices in the cereal producing areas of Norway [1]. Until 1960, most farms had domestic animals and produced cereals in rotation with pasture, potato or vegetables. Since then the Government Agricultural Policy (Oslo, Norway) has led to specialized cereal production in south-east and Central Norway. Severe epidemics caused by virulent, mycotoxigenic *F. graminearum* strains have occurred in both North America and Europe during the last 20 years [32,33,34]. Following the recent description of *F. langsethiae*, several authors have reported high levels of its mycotoxins T-2 and HT-2, especially in oats [24,26].

*Fusarium* species were recognized as cereal pathogens for many years before their production of mycotoxins was discovered. In Norway, research on diseases caused by members of this genus started more than seventy years ago [35]. During the last twenty years, greater awareness of the potential health problems caused by mycotoxins in humans and domestic animals has led to an almost exponential growth in research on mycotoxins and mycotoxin-producing fungi [2]. Roll-Hansen [14] in 1939, identified *F. graminearum* as the causal agent of a serious foot rot and head blight epidemic of oats in south-east Norway. In a report on diseases of field crops, Jørstad in 1945 [35] referred to *F. avenaceum* as the most common *Fusarium* spp. on cereals, and he diagnosed foot rot and head blight caused by *F. graminearum* in herbarium material of oats collected in 1911. The teleomorph stage, *Gibberella zeae* (Schwein.) Petch, was identified on barley and wheat obtained from Ullensaker, Akershus county (Norway) in 1941, and on wheat from Øyestad, Aust-Agder county (Norway) in 1939 [35]. Jørstad concluded that *F. avenaceum*, *F. culmorum*, *F. graminearum*, and *F. sporotrichioides* were the most common *Fusarium* species on Norwegian cereals [35].

Haave isolated *Fusarium* spp. from 416 plant samples of barley, oats and wheat during the years 1980–1983 [3] (Table 1). Symptoms of the plants included discoloration of the lower straw internodes, mycelium growth within the straw, whitehead or shriveled grain and occasionally pink spore layers in the culm. The most common *Fusarium* spp. were *F. culmorum*, followed by *F. avenaceum* and *F. graminearum*. During the dry summer of 1983, *F. culmorum* was isolated from 41% of the plant samples, while *F. avenaceum* was obtained from 22% of the samples. *F. culmorum* and *F. avenaceum* were more common on oats than on barley and wheat. Isolations from kernels during 1982–1983 yielded 19.2% *F. poae*, 5.8% *F. avenaceum*, 5.3% *F. culmorum*, and 2.3% *F. graminearum*.

Abbas *et al.* [36] isolated *Fusarium* spp. from soil samples collected in Finnmark county, Troms county and the cereal growing counties in central and south eastern Norway during 1985–1986. *Fusarium acuminatum* Ellis and Evereh. was most common in the north, while *F. avenaceum* was more uniformly distributed. Abbas *et al.* [37] determined mycotoxin production in the isolates and found that most of them did not produce trichothecenes. One *F. culmorum* isolate produced zearalenone. All but one of the *F. avenaceum* isolates produced fusarin C. Moniliformin was produced by most of the *F. acuminatum* isolates and half of the *F. avenaceum* isolates.

In a comparison of different tillage systems, Henriksen [4] isolated *Fusarium* spp. from cereal kernels harvested in field trials at five locations during the years 1994–1997 (Table 2). At Kvithamar Research Centre in Nord-Trøndelag county (Norway), *F. avenaceum* was found to be the dominant species in all four years. *F. poae* was the second most common species during 1997, while *F. graminearum* and *F. crookwellense* were the second most common species during 1995. At Apelsvoll Research Centre in Oppland county (Norway), *F. tricinctum*, *F. avenaceum* and *F. poae* were identified as the most common species. *F. avenaceum* was found to be the dominant *Fusarium* species at Brandval, Hedmark county (Norway). At Norderås, Akershus county (Norway), the Research Farm of the Norwegian University of Life Sciences *F. poae* and *F. avenaceum* were determined to be the most prevalent *Fusarium* species in the field during two of the four experimental years. At the Hauer farm, Frogn, Akershus county (Norway), *F. tricinctum* was identified as the dominant *Fusarium* spp. during two of the four years of field experiments [4].

Langseth and Rundberget [38] considered *F. avenaceum*, *F. culmorum*, *F. equiseti*, *F. graminearum*, *F. poae*, *F. sporotrichioides*, *F. torulosum* (Berk. and M.A. Curtis) Nirenberg and *F. tricinctum* to be the most common *Fusarium* species in Norwegian cereals, and mycotoxin production and cytotoxicity were determined in 34 isolates of these eight species.

In a post-harvest survey of wheat, barley and oat kernels from the major cereal-growing regions of Norway during 1994–1996, Kosiak *et al.* [5] identified *F. avenaceum*, *F. poae*, *F. tricinctum* and *F. culmorum* as the most common field fungi in all four regions where samples were taken (Table 3, Table 4 and Table 5). *F. graminearum*, *F. equiseti*, *F. sporotrichioides* and “powdery *F. poae*”, which was later described as *F. langsethiae*, were also common in all regions. Table 5 shows the occurrence of *Fusarium* spp. in oat samples from four regions. Kosiak *et al.* [5] provided data on the prevalence of *Fusarium* in barley and wheat (Table 3 and Table 4). The relatively high occurrence of *F. graminearum* in Central Norway was unexpected, since the temperature during most of the growing season is lower than in the cereal districts of south-east Norway. Samples from south-east Norway had a significantly lower total *Fusarium* prevalence than samples from the other regions. Wheat had lower *Fusarium* levels than barley and oats.

Henriksen and Elen [39] reported on *Fusarium* infections in wheat, barley and oat grain from field trials during 1996–1998. *Fusarium avenaceum* and *F. tricinctum* were the most common of the *Fusarium* spp. isolated. In oats, *F. tricinctum* was the dominant species during both the experimental years. In barley and wheat field experiments, *F. avenaceum* was the dominant species, while *F. culmorum*, *F. graminearum* and *F. tricinctum* were less frequently isolated from the harvested grain.

Halstensen *et al.* [40] used a PCR assay to identify *Fusarium* spp. in settled grain dust. The dominant species was *F. avenaceum*, which was identified in 94% of the grain dust samples from wheat, oats and barley harvested in 11 of the most important cereal-producing municipalities in Norway during 1999 and 2000. *Fusarium poae*, *F. langsethiae* and *F. culmorum* were also frequently detected in the grain dust samples. Less than 10% of the samples contained *F. graminearum* or *F. sporotrichioides*.

Based on the microbiological and chemical analyses of cereals grown in Norway, Uhlig *et al.* [41] identified *F. avenaceum* as the dominant species. They determined concentrations of up to 5.8 ppm enniatins in Norwegian grain harvested during the years 2000, 2001 and 2002, and concluded that conditions in Norway do not favor the production of moniliformin by *F. avenaceum*. Uhlig *et al.* [41] detected only low levels of moniliformin in Norwegian grain.

In parallel samples collected at organic and conventional farms during the years 2002–2004, Bernhoft *et al.* [42] identified *Fusarium* spp. from cereal grains. The mean percentages of kernels infected with the most common species, *F. avenaceum*, were 58% in organically grown barley and 56% in conventionally grown barley; in oats, the corresponding figures were 44% and 43%, and in wheat the percentages were 50% and 54%, respectively. The mean percentages of infected kernels of *F. graminearum* were 8% in organically grown barley and 10% in conventionally grown barley; in oats, the corresponding figures were 11% and 19%, and in wheat the percentages were 7% and 10%, respectively. *Fusarium poae* in oats was at mean proportions of 18% and 13%, respectively, while in barley and wheat this species was less common. The mean percentages of *F. culmorum*, *F. equiseti*, *F. langsethiae*, *F. sporotrichioides* and *F. tricinctum* were less than 10% in samples of barley, oats and wheat from the nine counties sampled.

Increased prevalence of *F. graminearum* and *F. langsethiae* has been reported from several north European countries. In Denmark, Nielsen *et al.* [34] used quantitative real-time PCR to identify *Fusarium* spp. During 2003–2007 DON was the dominant mycotoxin in wheat and *F. avenaceum*, *F. graminearum* and *F. culmorum* were the most prevalent species. In barley *F. poae*, *F. culmorum*, *F. langsethiae* and *F. graminearum* were most common*. Fusarium avenaceum*, *F. poae*, *F. langsethiae* and *F. graminearum* were the dominant species in oats. When Nielsen *et al.* [34] analyzed wheat and barley samples from 1957 to 1996 they found only low levels of *F. graminearum*, while *F. culmorum* was the dominant species. Yli-Mattila *et al.* [43] determined Fusarium DNA levels and mycotoxins in grain samples from Finland and Estonia. The correlation between *F. graminearum* DNA and DON levels was highly significant in both countries, while there was no correlation between *F. culmorum* DNA and DON levels. Yli-Mattila *et al.* [43] found no correlation between combined T-2 and HT-2 and combined *F. langsethiae*/*F. sporotrichoides* DNA levels. In the United Kingdom Opuko *et al.* [44] determined DNA of *F. langsethiae* and its mycotoxins in commercial cereal production. In oats *F. langsethiae* was the dominant *Fusarium* species, and high levels of its mycotoxins HT-2 and T-2 were detected in the harvested grains. DNA of *F. langsethiae* was not detected in roots or seedlings. Symptomless heads of oats, barley and wheat had high levels of HT-2 and T-2.

## 4. Conclusions

Mycotoxin producing fungi is a major problem in Norwegian cereal production. *Fusarium* species infect the cereal heads from anthesis to harvest. During the last decade this period has been wetter than normal, and severe *Fusarium* epidemics have developed in the cereal growing regions of the country. *Fusarium avenaceum* is still the most prevalent species, but the mycotoxins produced by *F. avenaceum* have low toxicity for humans and domestic animals. *Fusarium graminearum* has become the dominant DON producing species in Norwegian cereals, while *F. culmorum* has decreased in importance.

## References

[1] Statistics Norway. http://www.ssb.no/.

[2] Bernhoft A., Eriksen G.S., Sundheim L., Berntssen M., Brantsæter A.L., Brodal G., Fæste C.K., Hofgaard I.S., Rafoss T., Sivertsen T. (2013). Risk Assessment of Mycotoxins. in Cereal Grain in Norway. Opinion of the Scientific Steering Committee of the Norwegian Scientific Committee for Food Safety.

[3] Haave R. (1985). Prevalence and Pathogenicity of *Fusarium* Species on Cereals in Norway. Ph.D. Thesis.

[4] Henriksen B. (1999). Factors Affecting *Fusarium* Infection and Mycotoxin Content in Cereal Grains. Ph.D. Thesis.

[5] Kosiak B., Torp M., Skjerve E., Thrane U. (2003). The prevalence and distribution of *Fusarium* species in Norwegian cereals: A survey. Acta Agric. Scand. B.

[6] Leslie J.F., Summerell B.A. (2006). The *Fusarium* Laboratory Manual.

[7] Langseth W., Bernhoft A., Rundberget T., Kosiak B., Gareis M. (1999). Mycotoxin production and cytotoxicity of *Fusarium* strains isolated from Norwegian cereals. Mycopathologia.

[8] Thrane U. (1988). Screening for Fusarin C production by European isolates of *Fusarium* species. Mycotoxin Res..

[9] Schollenberger M., Drochner W., Müller H.-M. (2007). *Fusarium* toxins of the scirpentriol subgroup: A review. Mycopathologia.

[10] Covarerlli L., Beccari G., Steed A., Nicholson P. (2012). Colonization of soft wheat following infection of the stem base by *Fusarium culmorum* and translocation of deoxynivalenol to the head. Plant Pathol..

[11] Bakan B., Pinson L., Cahagnier B., Melcion D., Semon E., Richard-Molard D. (2001). Toxigenic potential of *Fusarium culmorum* strains isolated from French wheat. Food Addit. Contam..

[12] Miedaner T., Reinbrecht C. (2001). Trichothecene content of rye and wheat genotypes inoculated with a deoxynivalenol- and a nivalenol-producing isolate of *Fusarium culmorum*. J. Phytopathol..

[13] Hestbjerg H., Felding G., Elmholt S. (2002). *Fusarium culmorum* infection of barley seedlings: Correlation between aggressiveness and doxynivalenol content. J. Phytopathol..

[14] Roll-Hansen F. (1940). Undersøkelser av *Gibberella saubinetii* (Mont.) Sacc. som fotsyke på havre. Meld. Statens Frøkontroll.

[15] Oerke E.-C., Meier A., Dehne H.-W., Sulyok M., Krska R., Steiner U. (2010). Spatial variability of *Fusarium* head blight pathogens and associated mycotoxins in wheat crops. Plant. Pathol..

[16] Langseth W., Elen O. (1997). The occurrence of deoxynivalenol in Norwegian cereals—Differences between years and districts, 1988–1996. Acta Agric. Scand. B.

[17] Ward T.J., Bielawski J.P., Kistler H.C., Sullivan E., O’Donnell K. (2002). Ancestral polymorphism and adaptive evolution in the trichothecene mycotoxin gene cluster of phytopathogenic *Fusarium*. Proc. Natl. Acad. Sci. USA.

[18] Yli-Mattila T. (2010). Ecology and evolution of toxigenic *Fusarium* species in cereals in Northern Europe and Asia. J. Plant Pathol..

[19] Desjardins A.E., Proctor R.H., Bai G., McCormick S.P., Shaner G., Buechley G., Hohn T.M. (1996). Reduced virulence of trichothecene-nonproducing mutants of *Gibberella zeae* in wheat field tests. Mol. Plant Microbe Interact..

[20] Waalwijk C., Kastelein P., de Vries I., Kerényi Z., van der Lee T., Hesselink T., Köhl J., Kema G. (2003). Major changes in *Fusarium* spp. in wheat in the Netherlands. Eur. J. Plant Pathol..

[21] Xu X.M., Nicholson P., Thomsett M.A., Simpson D., Cooke B.M., Doohan F.M., Brennan J., Monaghan S., Moretti A., Mule G. (2008). Relationship between the fungal complex causing *Fusarium* head blight of wheat and environmental conditions. Phytopathology.

[22] Torp M., Nirenberg H.I. (2004). *Fusarium langsethiae* sp. nov. on cereals in Europe. Int. J. Food Microbiol..

[23] Knutsen A.K., Torp M., Holst-Jensen A. (2004). Phylogenetic analyses of the *Fusarium poae*, *Fusarium sporotrichoides* and *Fusarium langsethiae* species complex based on partial sequences of translation elongation factor-1 α gene. Int. J. Food Microbiol..

[24] Imathiu S.M., Ray R.V., Back M., Hare M.C., Edwards S.G. (2009). *Fusarium langsethiae* pathogenicity and aggressiveness towards oats and wheat in wounded and unwounded *in vitro* detached leaf assays. Eur. J. Plant Pathol..

[25] Divon H.H., Razzaghian J., Udnes-Aamot H., Klemsdal S.S. (2011). *Fusarium langsethiae* (Torp and Nirenberg) investigation of alternative infection routes in oats. Eur. J. Plant Pathol..

[26] Thrane U., Adler A., Clasen P.-E., Galvano F., Langseth W., Lew H., Logrieco A., Nielsen K.F., Ritieni A. (2004). Diversity in metabolite production by *Fusarium langsethiae*, *Fusarium poae*, and *Fusarium sporotrichoides*. Int. J. Food Microbiol..

[27] Aamot H., Hofgaard I.S., Brodal G., Elen O., Holen B., Klemsdal S.S. (2013). Evaluation of rapid test kits for quantification of HT-2 and T-2 toxins in naturally contaminated oats. World Mycotoxin J..

[28] Liu W., Sundheim L. (1996). Nitrate nonutilizing mutants and vegetative compatibility groups in *Fusarium poae*. Fungal Genet. Biol..

[29] Liu W., Sundheim L., Langseth W. (1997). Trichothecene production and relationship to vegetative compatibility groups in *Fusarium poae*. Mycopathologia.

[30] Yli-Mattila T., Mach R., Alekhina I.A., Bulat S.A., Koskinen S., Kullnig-Gradinger C.M., Kubicek C.P., Klemsdal S.S. (2004). Phylogenetic relationship of *Fusarium langsethiae* to *Fusarium poae* and *Fusarium sporotrichoides* as inferred by IGS, ITS, β-tubulin sequences and UP-PCR hybridization analysis. Int. J. Food Microbiol..

[31] Golinski P., Perkowski J., Kostecki M., Grabarkiewicz-Szezesna J., Chelkowski J. (1996). *Fusarium* species and *Fusarium* toxins in wheat in Poland: A comparison with neighbour countries. Sydowia.

[32] Miller J.D. (2008). Mycotoxins in small grain: Old problems, new challenges. Food Addit. Contam..

[33] Trail F. (2009). For blighted waves of grain: *Fusarium graminearum* in the postgenomic era. Plant Physiol..

[34] Nielsen L.K., Jensen J.D., Nielsen G.C., Jensen J.E., Spliid N.H., Thomsen I.K., Justesen A.F., Collinge D.B., Jørgensen L.N. (2011). *Fusarium* head blight of cereals in Denmark: Species complex and related mycotoxins. Phytopathology.

[35] Jørstad I. (1945). Parasittsoppene på kultur- og nyttevekster i. Norge I. Sekksporesopper (Ascomycetes) og konidiesopper (Fungi imperfecti). Meld. Statens Plantepatol. Inst..

[36] Abbas H.K., Mirocha C.J., Berdal B.P., Sundheim L., Gunther R., Johnsen B. (1987). Isolation and toxicity of *Fusarium* species from various areas of Norway. Acta Agric. Scand..

[37] Abbas H.K., Mirocha C.J., Gunther R. (1989). Mycotoxins produces by *Fusarium* isolates obtained from agricultural and nonagricultural areas (Arctic) of Norway. Mycopathologia.

[38] Langseth W., Rundberget T. (1999). The occurrence of HT-2 toxin and other trichothecenes in Norwegian cereals. Mycopathologia.

[39] Henriksen B., Elen O.N. (2005). Natural *Fusarium* grain infection level in wheat, barley and oat after early application of fungicides and herbicides. J. Phytopathol..

[40] Halstensen A.S., Nordby K.-C., Klemsdal S.S., Elen O., Clasen P.-E., Eduard W. (2006). Toxigenic *Fusarium* spp. as determinants of trichothecene mycotoxins in settled grain dust. J. Occup. Environ. Hyg..

[41] Uhlig S., Jestoi M., Parikka P. (2007). *Fusarium Avenaceum*—The North European situation. Int. J. Food Microbiol..

[42] Bernhoft A., Clasen P.-E., Kristoffersen A.B., Torp M. (2010). Less *Fusarium* infestation and mycotoxin contamination in organic than in conventional cereals. Food Addit. Contam..

[43] Yli-Mattila T., Rämö S., Tanner R., Loiveke H., Hietaniemi V. (2011). *Fusarium* DNA as compared to mycotoxin levels in Finnish and Estonian grain samples. Plant. Breed. Seed Sci..

[44] Opoku N., Back M., Edwards S.G. (2013). Development of *Fusarium langsethiae* in commercial cereal production. Eur. J. Plant Pathol..

